# Adipose-derived stem cells alleviate liver injury induced by type 1 diabetes mellitus by inhibiting mitochondrial stress and attenuating inflammation

**DOI:** 10.1186/s13287-022-02760-z

**Published:** 2022-04-01

**Authors:** Yanli Hou, Wenyu Ding, Peishan Wu, Changqing Liu, Lina Ding, Junjun Liu, Xiaolei Wang

**Affiliations:** 1 Endocrine and Metabolic Diseases Hospital of Shandong First Medical University, Shandong First Medical University and Shandong Academy of Medical Sciences, Jinan, China; 2 Shandong First Medical University, Jinan, China

**Keywords:** Type 1 diabetes mellitus, Liver injury, Adipose-derived stem cells, Mitochondrial stress, Inflammation, Immune regulation

## Abstract

**Background:**

Type 1 diabetes mellitus (T1D) is a worldwide health priority due to autoimmune destruction and is associated with an increased risk of multiorgan complications. Among these complications, effective interventions for liver injury, which can progress to liver fibrosis and hepatocellular carcinoma, are lacking. Although stem cell injection has a therapeutic effect on T1D, whether it can cure liver injury and the underlying mechanisms need further investigation.

**Methods:**

Sprague–Dawley rats with streptozotocin (STZ)-induced T1D were treated with adipose-derived stem cell (ADSC) or PBS via the tail vein formed the ADSC group or STZ group. Body weights and blood glucose levels were examined weekly for 6 weeks. RNA-seq and PCR array were used to detect the difference in gene expression of the livers between groups.

**Results:**

In this study, we found that ADSCs injection alleviated hepatic oxidative stress and injury and improved liver function in rats with T1D; potential mechanisms included cytokine activity, energy metabolism and immune regulation were potentially involved, as determined by RNA-seq. Moreover, ADSC treatment altered the fibroblast growth factor 21 (FGF21) and transforming growth factor β (TGF-β) levels in T1D rat livers, implying its repair capacity. Disordered intracellular energy metabolism, which is closely related to mitochondrial stress and dysfunction, was inhibited by ADSC treatment. PCR array and ingenuity pathway analyses suggested that the ADSC-induced suppression of mitochondrial stress is related to decreased necroptosis and apoptosis. Moreover, mitochondria-related alterations caused liver inflammation, resulting in liver injury involving the T lymphocyte-mediated immune response.

**Conclusions:**

Overall, these results improve our understanding of the curative effect of ADSCs on T1D complications: ADSCs attenuate liver injury by inhibiting mitochondrial stress (apoptosis and dysfunctional energy metabolism) and alleviating inflammation (inflammasome expression and immune disorder). These results are important for early intervention in liver injury and for delaying the development of liver lesions in patients with T1D.

**Supplementary Information:**

The online version contains supplementary material available at 10.1186/s13287-022-02760-z.

## Background

Diabetes mellitus, characterized by chronic hyperglycemia, refers to a group of clinical syndromes caused by both genetic and environmental factors and has become a critical health concern worldwide owing to its high prevalence [1, 2]. Over the past 30 years, the prevalence of diabetes in China has soared from 0.67% in 1980 to 12.8% in 2017 [3]. The complications of diabetes, such as diabetic nephropathy and retinopathy, have a high rate of disability and mortality [4]; moreover, recent studies have suggested that liver alterations constitute another diabetic complication [5]. Hyperglycemia caused by diabetes increases the risk of liver injury and fibrosis, which, in the absence of timely and early intervention, will progress to irreversible cirrhosis and liver cancer [6], seriously affecting patient health. Type 1 diabetes mellitus (T1D) is closely associated with liver disease [5, 7], but the pathophysiological basis and evolution of liver alterations in T1D are still not fully clear, and effective early interventions are lacking.

Patients with T1D are inevitably treated with insulin on a continuous basis; however, some supportive therapies to control blood glucose levels and minimize adverse reactions to insulin are needed. Cell therapy has become a recent research focus and has been recently applied; furthermore, stem cells have been used in the treatment of T1D because of their regenerative and immunomodulatory potential [8]. Studies have shown that adipose-derived stem cell (ADSC) injection into the tail vein alleviates renal injury and promotes podocyte repair in diabetic rats [9] and protects against acute liver failure [10] and liver regeneration in animals [11]. Nevertheless, the role of ADSCs in diabetic liver injury has not been studied.

The liver is rich in mitochondria, and mitochondrial stress and dysfunction play a significant role in fatty liver, liver fibrosis, viral hepatitis and other liver diseases. By activating membrane lipid peroxidation to change the function of biofilms, covalently binding biomacromolecules and destroying enzyme activity, mitochondrial stress causes different degrees of liver injury and apoptosis. In this process, cytokines such as tumor necrosis factor α (TNF-α) and transforming growth factor β (TGF-β) and nuclear factor-κB (NF-κB) pathway, also act together with mitochondrial stress [12]. TNF-α can active the canonical NF-κB pathway to promote expression of other proinflammatory genes and has fundamental roles in inflammation and host defense [13, 14]. TGF-β1, one of the most important cytokines, has been found to promote fibrosis [12]. Mitochondria, the "energy factories" of cells, play an important role in regulating energy metabolism. AMPK is a well-characterized cellular energy sensor. The AMPK signaling pathway is involved in various types of energy metabolism and the pathogenesis of mitochondrial diseases, including oxycholic acid-induced liver injury [15], renal ischemia/reperfusion injury [16] and diabetic peripheral neuropathy [17]. AMPK-dependent Ca^2+^ signaling in mitochondria plays an important role in mitosis during acute cell stress [18]. Mitochondrial stress plays an important role in oxidative stress in liver cells. Mitochondrial dysfunction can lead to liver oxidative stress and lipid peroxidation, excessive cytokine release and apoptosis and is essential for the development and progression of liver disease [19–22].

The periodic opening and closing of the mitochondrial permeability transition pore (mPTP), a nonselective channel in the mitochondrial inner membrane, under physiological conditions are important for the maintenance of cell homeostasis. Excessive irreversible opening of the mPTP [23, 24] causes mitochondrial swelling and dysfunction of the mitochondrial respiratory chain (mainly impaired activity of mitochondrial complex enzymes I-V), eventually leading to mitochondrial stress and energy metabolism disorders [25]. Our previous study confirmed that mitochondrial stress induced by excessive mPTP opening plays an important role in hepatic insulin resistance [26]. Nevertheless, the specific mechanism by which mPTP opening affects liver injury in T1D is unclear.

Liver lesions in type 2 diabetes mellitus (T2D) are closely related to changes in nonalcoholic fatty liver disease. The progression and pathogenesis of this disease from lipid deposition to cirrhosis have been extensively studied [27, 28]. Although the rat model of T1D induced by streptozotocin (STZ) provides important information for the study of liver injury [29, 30], the pathogenesis and treatment of hepatic lesions in T1D remain poorly studied. Therefore, this study aimed to explore the pathogenesis of liver injury in T1D and investigate the protective effect of ADSCs.

## Methods

### Isolation and characterization of ADSCs

ADSCs were isolated from Sprague–Dawley (SD) male rats at 5 ~ 6 weeks of age. SD rats were purchased from Jinan Pengyue Laboratory Animal Breeding Co., Ltd. (Shandong, China). Briefly, white adipose tissue from the inguinal region of the rats was digested with collagenase I (Solarbio, China) at 37 °C for 1 h with continuous shaking. The reaction was stopped with the addition of culture medium, and the material was pelleted via centrifugation for 10 min at 1200 rpm. The acquired cells were resuspended and filtered through 100-μm filters. After being washed twice, the cells were cultured in DMEM containing 1 g/L glucose (Gibco), 10% FBS (Gibco) and 1% penicillin/streptomycin at 37 °C and 5% CO_2_, and the medium was replaced every 3 days. ADSCs at passage 3 were characterized by the adipogenic and osteogenic differentiation potential, and surface markers (the data were not shown). ADSCs between passage 3 and 5 were used in the next animal and cell experiments.

### Animal experiment

The experimental protocols were approved by the Research Ethics Committee of the Shandong Institute of Endocrine & Metabolic Diseases.

Eight-week-old male SD rats were housed in colony cages with a 12-h light/dark cycle under constant temperature and humidity and given free access to water and normal food. All rats had similar body weights and growth statuses before STZ administration. Twenty rats were fasted for 16 h (with free access to water) and were then intraperitoneally injected with 60 mg/kg STZ (Solarbio, China). The fasting blood glucose levels from samples taken from the tail tip were examined daily 3 days after STZ injection using a glucometer. Rats with a blood glucose level higher than 16.7 mmol/l measured twice were diagnosed with high-dose STZ-induced diabetes. That day was referred to as day 0 of experiment.

On day 14 after STZ-induced diabetes establishment, 16 diabetic rats were randomized into 2 groups. Rats treated with one dose of 2.0 × 10^6^ ADSCs (passages 3 ~ 5) via the tail vein formed the ADSC-treated (ADSC) group, and the other group was comprised of rats injected with the same dose of PBS (STZ group). An additional 8 normal rats served as the normal control (NC) group. Body weights and blood glucose levels were examined weekly. After 6 weeks of ADSC or PBS treatment, all rats were anesthetized for the harvesting of blood from the jugular vein after fasting, followed by killing.

### RNA-Seq analysis

Total RNA was extracted from liver tissue using a TRIzol® Reagent Kit (Thermo Fisher Scientific, USA) according to the manufacturer's instructions. The total RNA concentration was measured with a NanoDrop instrument, and the OD260/OD280 ratio was used as an index of RNA purity. Agarose gel electrophoresis was used to measure RNA integrity and gDNA contamination.

RNA-seq analysis was performed by WeHealth Biotech (Shanghai, China). A sequencing library was built with the NEBNext® Ultra™ II Directional RNA Library Prep Kit, and the sequences were verified and quantified by agarose gel electrophoresis. The Illumina HiSeq X platform was used to carry out 150-bp two-terminal sequencing. The expression level of each gene was estimated by counting the sequences (reads) mapped to the reference sequence, with reads per kilobases per million reads (RPKM), the most commonly used method, used to estimate the gene expression level. The DEGs were analyzed using the MA plot-based method with a random sampling (MARS) model in the DEGseq v1.20.0 package. Differences in gene expression among samples were considered significant when all of the following conditions were met: fold change > 2 or < 0.5, FDR (q value) < 0.05 and RPKM > 2 for at least one sample.

To explore gene function and the regulatory networks involved in the rat liver, DEGs were subjected to GO functional enrichment analysis and KEGG metabolic pathway mapping.

### ELISA

Liver FGF21, TGF-β1 and AGE levels were measured with commercial ELISA kits (Boster Biotech, China and OmnimAbs, USA, respectively) according to the manufacturer’s protocols, and the concentrations were normalized to the corresponding protein level.

### Isolation of mitochondria

Mitochondria were isolated from the livers with a previously indicated protocol with modifications [24]. The experimental procedure used was described in detail in our previous work [31].

### Assessment of mitochondrial swelling

Freshly isolated liver mitochondria (20 μg) were suspended in 200 μl of swelling assay buffer (in mmol/L: KCl 150, HEPES 5, K_2_HPO_4_ 2, glutamate 5, malate 5, pH 7.2), and swelling was stimulated with calcium (1 μmol/mg protein), after which the absorbance at 540 nm was measured on a SpectraMax M2/M2e microplate reader (Molecular Devices, LLC).

### Transmission electron microscopy (TEM)

Liver tissues were pretreated as described in our previous study [26]. Briefly, liver tissues were fixed for 2 h with 2.5% glutaraldehyde in 0.1 M cacodylate buffer, washed three times with 0.1 M cacodylate buffer and fixed in 1% osmium tetroxide for 1 h. The samples were then examined with a transmission electron microscope (JEM-1200EX, JEOL Co., Japan).

### Histology and serological index measurements

For histology, tissue blocks were cut into sections and subjected to H&E staining. Serological index kits were purchased from TECON Biotech (Ningbo, China).

### PCR array

The rat RT2 Profiler PCR array was used to analyze RNA extracted from the livers of rats in the STZ and ADSC groups. One microgram of purified RNA was used for amplification to produce cDNA using the RT2 First Strand Kit (QIAGEN). Rat mitochondria (QIAGEN, PARN-087Z) and inflammasome (QIAGEN, PARN-097Z) PCR arrays were used to analyze the samples. The 384-well plate array was performed using a LightCycler system (Roche) according to the manufacturer’s instructions. The obtained data were interpreted using the SABiosciences PCR Array data analysis web portal. Quality control of all PCR arrays was carried out by assessing the quality of the internal controls: the reverse transcription control (RTC) and positive PCR control (PPC). Any array for which the difference between two values was < 5 passed the quality control step; all arrays passed the quality control test. Variations between groups are defined as fold changes, and genes whose expression was significantly changed (fold change was 2 or above).

### L02 cell culture

L02 cells were maintained in RPMI 1640 medium (Gibco) supplemented with 10% FBS (Gibco). After the cells grew to approximately 60 ~ 80% confluence, the medium was replaced with medium supplemented with 2% FBS. The cells were treated with high glucose (HG,25 mM) and advanced glycation end products (AGEs, BioVision) for the indicated duration, and the corresponding control group was treated with BSA as a control (BioVision). L02 cells were treated with ADSC supernatant to determine the effect of ADSCs on HG- and AGEs-induced mitochondrial stress and apoptosis.

### Flow cytometry (FCM)

The L02 cells were collected, and staining was performed according to the method provided by the manufacturer of an Annexin V-FITC/PI apoptosis detection kit (Solarbio, China). The samples were analyzed by FCM and evaluated based on the percentage of cells that were positive for Annexin V.

### In situ detection of mitochondrial ROS

The cells were washed three times, incubated with MitoSox Red (1:2000, Invitrogen) at 37 °C for 30 min and then washed three times. The entire process was carried out rapidly and gently. Then, we examined the cells under a Delta Vision Ultra cell imaging system (GE). Images were analyzed, and fluorescence intensity was quantified using ImageJ software as described in a previous report [32].

### Western blotting

To obtain whole-protein extracts, L02 cells or rat liver was lysed in lysis buffer containing a protease inhibitor. Protein detection was performed using a FluorChem Q system (Cell Biosciences). Densitometric analysis of the scanned blots was carried out using ImageJ software, and the results are expressed as the fold change relative to the control after normalization to the respective internal control. Antibodies against CypD, Sod2, GPX4, COXIV and β-actin obtained from Abcam and Proteintech, respectively, were used for western blotting.

### JC-1 staining

The L02 cells were treated and stained using a commercial kit (Beyotime, Shanghai, China). Red fluorescence was emitted by JC-1 aggregates in healthy mitochondria with polarized inner mitochondrial membranes, while green fluorescence was emitted by cytosolic JC-1 monomers and indicated membrane potential dissipation. Merged images indicated the colocalization of JC-1 aggregates and monomers.

### Statistical analyses

Statistical analyses were performed using SPSS software. The results are reported as the mean ± SD. Comparisons between different groups were performed using one-way ANOVA followed by LSD test (equal variances assumed) or Dunnett's T3 test (equal variances not assumed). Differences with a two-tailed *p* < 0.05 were considered significant.

## Results

### Diabetic rat model and physiological differences

**Fig. 1:**
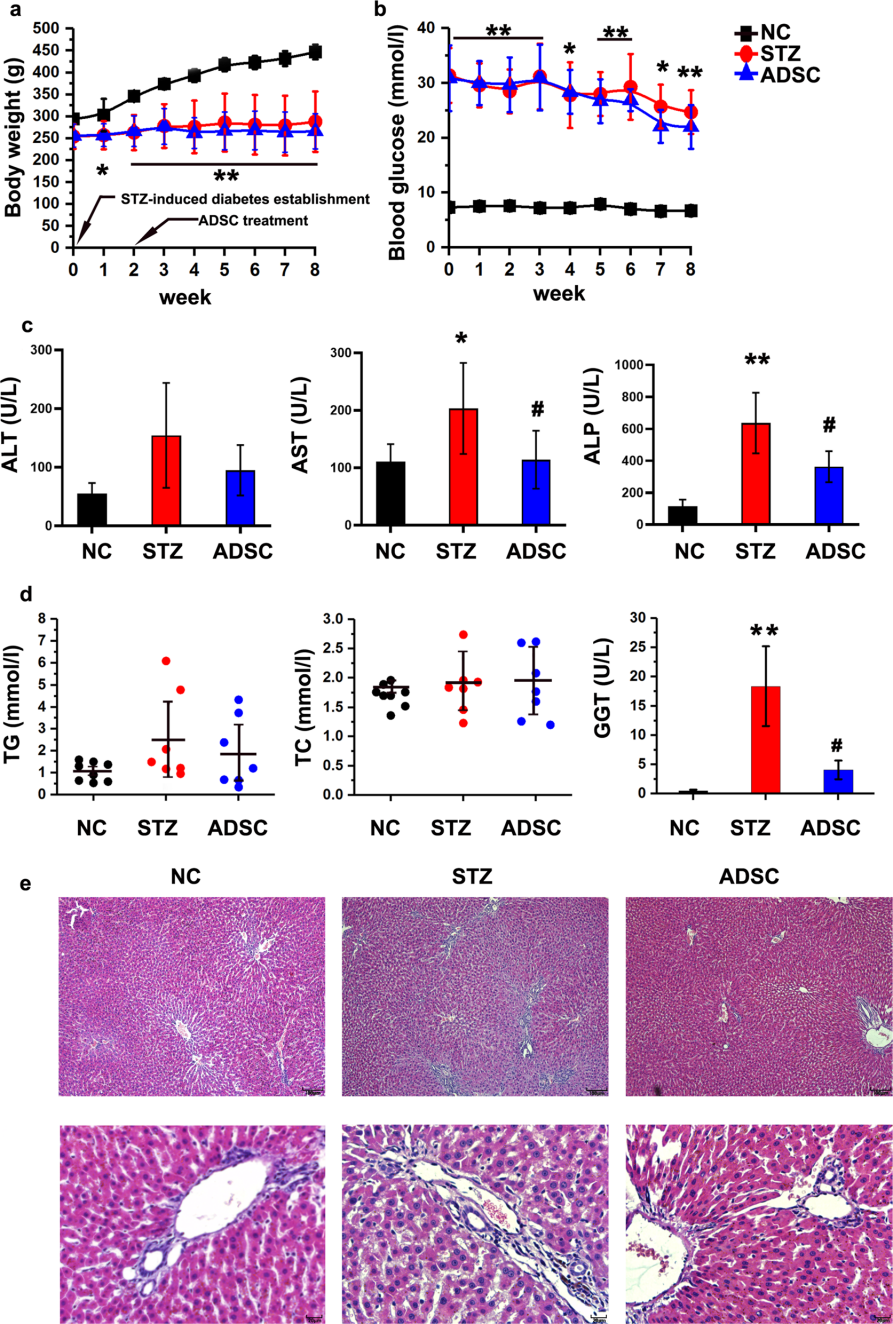
Physiological differences between the three groups. **a**, **b** Body weight and blood glucose levels of rats were examined weekly. The day that STZ-induced diabetic rats were established was referred to as day 0 of the experiment. ADSCs were injected at the end of the second week. **c**, **d** Serological index. **e** H&E staining (the upper panel is 10X, and the lower panel is 40X). *NC* normal control group, *STZ* STZ-induced diabetic group, *ADSC group* ADSC-treated group. *p < 0.05 and **p < 0.01 versus the NC group, #*p* < 0.05 versus the STZ group

The body weights and blood glucose levels of rats in the NC, STZ and ADSC groups are shown in Fig. 1a, b. Compared to the NC group, the STZ group showed significant changes in body weight and fasting blood glucose. These data indicated that we successfully established a diabetic rat model. To observe the long-term effects of ADSCs on liver damage, we treated the diabetic rats with ADSCs for 6 weeks and collected specimens at the end. Serological tests showed that the alanine aminotransferase (ALT), aspartate aminotransferase (AST) and alkaline phosphatase (ALP) levels were increased in the STZ group and decreased in the ADSC group, implying the protective effect of ADSCs on liver function and injury (Fig. 1c). ADSC treatment reduced the increases in serum triglyceride (TG) and γ-glutamyl transpeptidase (GGT) levels induced by STZ but had no significant effect on total cholesterol (TC) (Fig. 1d). Because GGT is a key indicator of oxidative stress in the liver [33], the results suggest that ADSC treatment may alleviate hepatic oxidative stress injury. Histological analysis of the liver by hematoxylin–eosin (H&E) staining (Fig. 1e) showed that STZ could cause infiltration of inflammatory cells, hyperplasia of fibers and proliferation of the bile duct in the hepatic portal area. ADSC treatment reduced inflammatory infiltration and thickening of the vascular wall in the portal area induced by STZ.

### RNA sequencing (RNA-Seq) analysis of liver tissue

#### Identification of differentially expressed genes (DEGs)

**Fig. 2:**
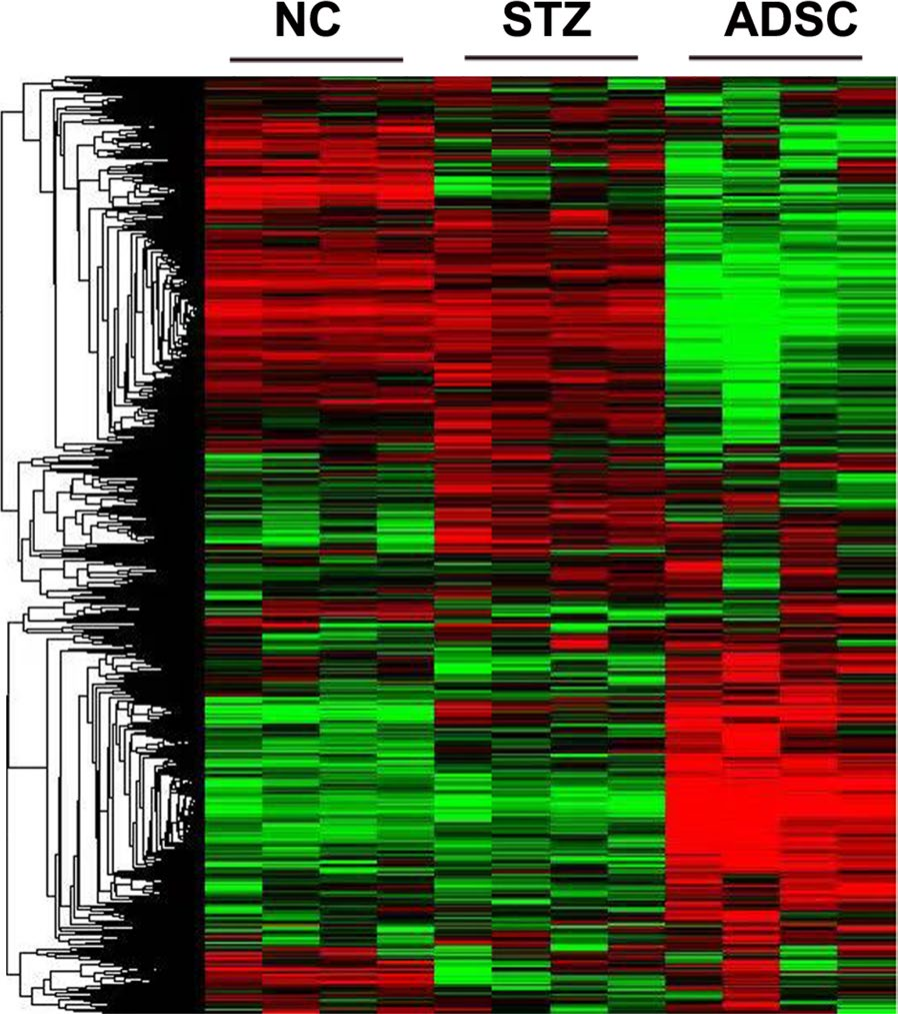
Differential gene change heat map of RNA-seq analysis. Each row represents a transcript, and each column represents a sample. Red represents upregulation of gene expression, and green represents downregulation of gene expression

RNA-seq was performed to identify differences between the three groups. To assess the transcriptome sequencing results, a fold change > 2 or < 0.5 and an FDR (q value) < 0.05 were used to screen DEGs. The heat map of the DEGs prepared by hierarchical clustering is shown in Fig. 2. The screening results identified 486 DEGs in the STZ group compared with the NC group and 2132 DEGs in the ADSC group compared with the STZ group (Additional file 1: Fig. S1).

#### Gene ontology (GO) functional enrichment analysis of the DEGs

**Fig. 3:**
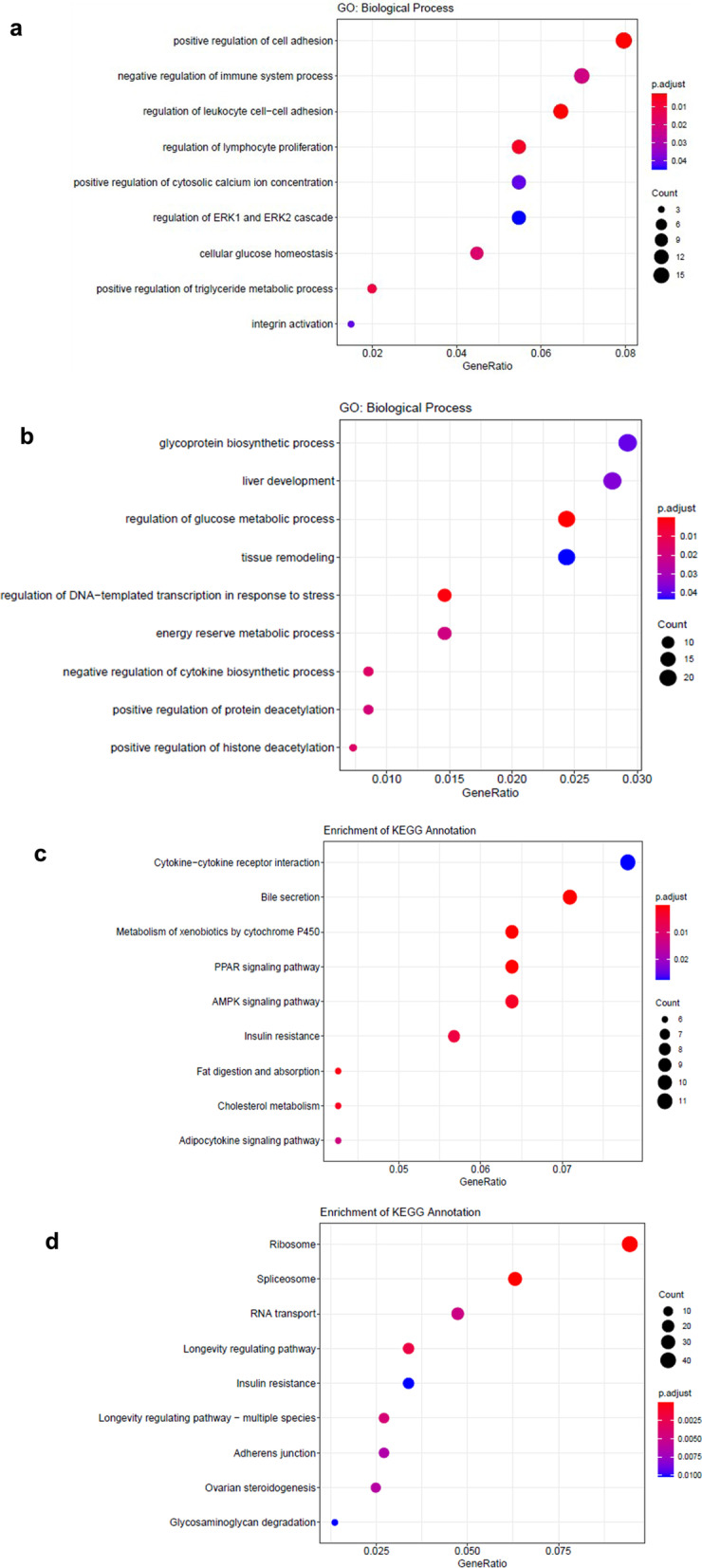
GO enrichment (**a**, **b**) and KEGG pathway enrichment (**c**, **d**) analysis of DEGs. **a**, **c** Represent the STZ group compared with the NC group. **b**, **d** Represent the ADSC group compared with the STZ group

To clarify the functions of the DEGs, GO functional enrichment analysis was performed. Nine GO categories were selected according to their q-values, and the functions of the DEGs were assessed by the corresponding GO annotations. We found obvious differences in the expression of genes related to the biological processes in liver injury and liver fibrosis (such as lymphocyte proliferation, cell adhesion, ERK1/2 regulation, immune regulation and integrin activation) in the STZ group compared with the NC group (Fig. 3a). In terms of molecular function, significant differences in the expression of genes related to cytokine activity were observed. Studies have shown that ADSCs can secrete a certain number of cytokines. Our results showed that in the ADSC group compared with the STZ group, the expression of genes involved in energy metabolism, glycoprotein biosynthesis, stress, protein deacetylation and other biological processes was significantly different, in addition to the expression of cytokines (Fig. 3b).

#### Kyoto Encyclopedia of Genes and Genomes (KEGG) classification of the DEGs

To identify the pathogenic mechanism of T1D and molecular pathways involved in the effects of ADSCs on the liver, DEGs from the three groups were mapped with the KEGG pathway database. Nine most enriched pathways are shown in Fig. 3. When the STZ group was compared with the NC group, we found significant differences in the expression of genes related to the cytokine–cytokine receptor interaction, the AMPK signaling pathway, the PPAR signaling pathway and insulin resistance (Fig. 3c). When the STZ group ADSC group was compared, significant differences in the expression of genes involved in insulin resistance, longevity, RNA transport and so on were observed (Fig. 3d).

RNA-seq results showed that the expression of fibroblast growth factor 21 (FGF21) was decreased in the STZ group compared with the NC group. Vascular endothelial growth factor (VEGF-α) expression was increased, and Tgfbr3 and Tgfbr2 expression was decreased in the ADSC group compared with the STZ group (data not shown), providing valuable information for further studies on the underlying molecular mechanisms. The results of GO classification and KEGG enrichment analysis indicated that disordered immune regulation and liver injury may have occurred during the pathogenesis of STZ-induced diabetes in rats and that ADSC treatment may affect cytokine activity and energy metabolism.

### ADSC treatment alleviated liver injury and mitochondrial stress in diabetic rats

**Fig. 4:**
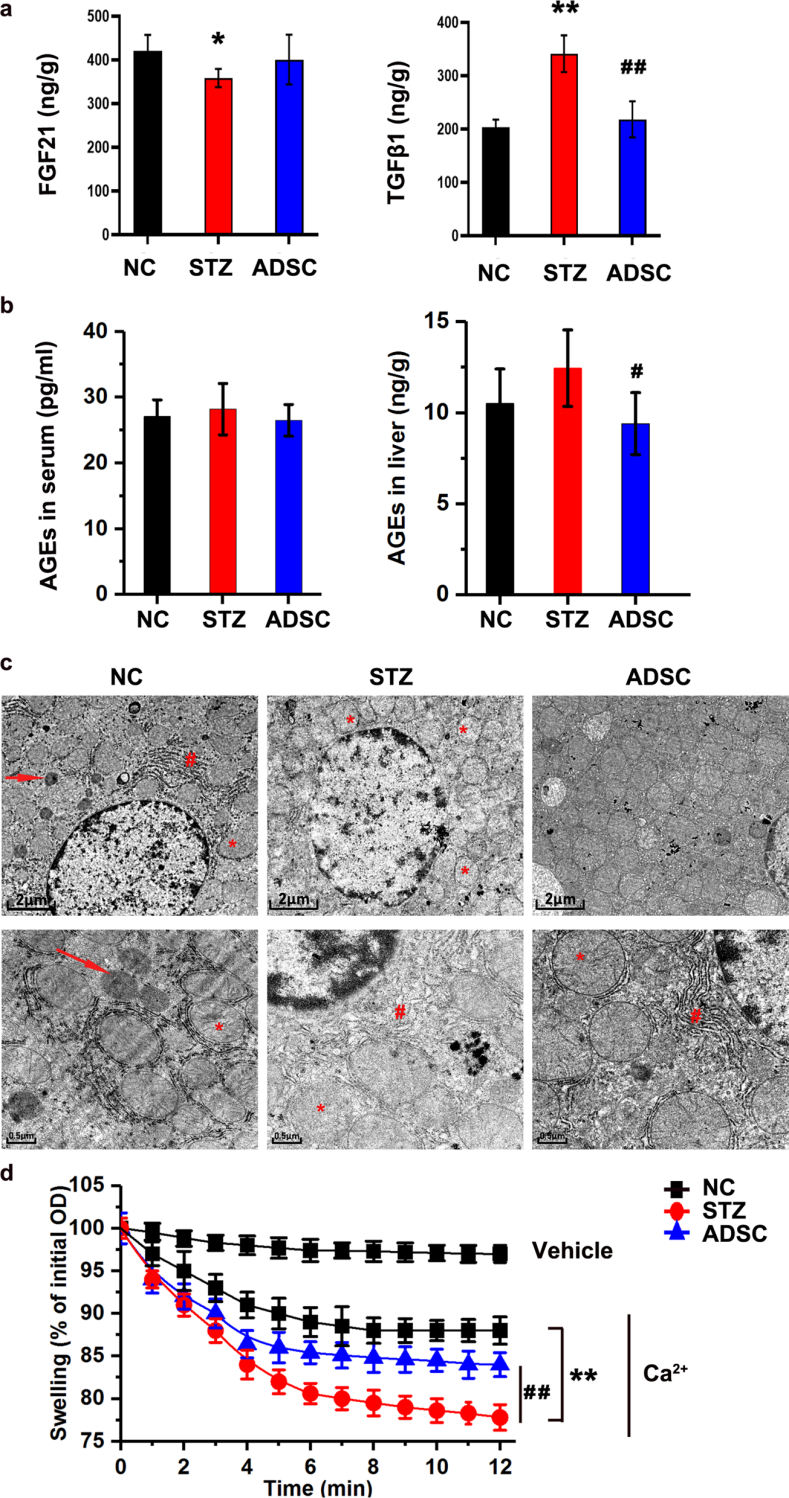
Effects of ADSCs on liver injury and hepatic mitochondrial stress in vivo*.* **a** FGF21 and TGF-β1 levels in the liver. **b** AGEs in the serum and livers. **c** TEM analysis (the upper is 7500X and the lower is 20000×). The accumulation of lipid droplets, marked by the arrow in red, was decreased in the diabetic rats compared to the NC group. TEM images of liver sections from the STZ group showed mitochondrial deformation and vacuolization, swollen mitochondria with disrupted cristae and dilation of the endoplasmic reticulum, while representative images of the ADSC group illustrated the hepatoprotective effect of ADSC treatment. Mitochondria were marked by “*”, and endoplasmic reticulum was marked by “#”. **d** Mitochondrial swelling test. Liver mitochondria were isolated, and Ca^2+^ (1 µmol/mg protein)-induced mitochondrial swelling was measured and expressed as the percentage decrease in the initial optical density (OD) at an absorbance of 540 nm. The severity of swelling is represented by the slope of the curve. *p < 0.05 and **p < 0.01 versus the NC group, #*p* < 0.05 and ##*p* < 0.01 versus the STZ group

Based on the above RNA-seq results, ① we observed changes in FGF21 and TGF-β1 in the liver. FGF21, which is mainly expressed in the liver, plays an important role in regulating stress and glycolipid metabolism. TGF-β1 was significantly increased at mRNA level in a liver fibrosis model [34, 35]. ELISA results (Fig. 4a) showed that ADSC treatment decreased TGF-β levels, suggesting that ADSC treatment alleviates liver injury in diabetic rats. ② To assess the glycoprotein biosynthetic process, we measured the levels of advanced glycation end products (AGEs) in the serum and livers of the rats (Fig. 4b). The production of AGEs is an important protein glycosylation process in the body in which a series of complex reactions with reducing sugars (such as glucose and fructose) and free amino groups form AGEs. Hyperglycemia can increase the level of endogenous AGEs in the body, and islet cell inflammation and diabetic nephropathy are related to the accumulation of AGEs [36–40]. Our results showed that the serum AGE levels in each group were not significantly different, but ADSC treatment inhibited the increase in hepatic AGEs induced by STZ (Fig. 4b), suggesting that ADSC treatment may have a protective effect in the liver by inhibiting the production of AGEs.

RNA-seq analysis revealed that genes involved in energy reserves, metabolic processes, the AMPK pathway and stress were significantly differentially expressed among the three groups. The liver is rich in mitochondria, and mitochondrial stress plays an important role in oxidative stress in liver cells. Based on our previous finding that ADSC treatment could reduce the increase in GGT, an oxidative stress marker of liver cells, we speculated that ADSCs would inhibit mitochondrial stress in the livers of diabetic rats. First, we observed the morphology of the liver. Representative TEM images (Fig. 4c) of liver sections obtained from the NC group show normal hepatocyte architecture. The nuclei were round in shape, and the cytoplasm contained many organelles, particularly rough endoplasmic reticulum and mitochondria. Lipid droplets were found. TEM images of liver sections from the STZ group showed mitochondrial deformation and vacuolization, swollen mitochondria with disrupted cristae and dilation of the endoplasmic reticulum, while representative images of the ADSC group illustrated the hepatoprotective effect of ADSC treatment. Because the integrity of the mitochondrial structure has a direct impact on mitochondrial function, we then observed the degree of mitochondrial swelling, an index that reflects mPTP opening and mitochondrial function [24]. The hepatic mitochondria in the STZ group showed greater swelling in response to Ca^2+^ than those in the NC group, but this difference was decreased by ADSC treatment (Fig. 4d), implying the role of ADSCs in protecting hepatic mitochondrial function.

Mitochondria are the main sites of reactive oxygen species (ROS) production, and excessive mPTP opening results in mitochondrial ROS generation. The overproduction of ROS to a degree exceeding the ability to remove them results in mitochondrial stress, leading to disordered energy metabolism, damage and even cell apoptosis [41, 42]. We treated L02 hepatocytes with high glucose (HG) and AGEs to mimic our rat model and analyzed cellular apoptosis, mitochondrial stress and the protective effect of the ADSC supernatant. First, FCM was used to analyze cell apoptosis. Cells were treated with HG and 100 µg/ml or 200 µg/ml AGEs for 48 h, and the results showed that HG + AGEs did not induce significant apoptosis (Additional file 2: Fig. S2a). JC-1 is an important indicator of early cell apoptosis and a marker of mitochondrial stress. Second, we treated cells with HG + AGEs (100 µg/ml) for 24 h or 48 h, and JC-1 staining showed that ADSC treatment moderated the decrease in mitochondrial membrane potential induced by HG + AGEs at 24 h in a time-dependent manner (Additional file 2: Fig. S2b). To evaluate mitochondrial ROS generation, we then stained hepatocytes with MitoSox Red, a unique fluorogenic dye that allows the selective detection of superoxide production in mitochondria. The results showed that ADSCs reduced the increase in mitochondrial ROS production caused by HG + AGEs in hepatocytes (Additional file 2: Fig. S2c).

Cyclophilin D (CypD), located in the mitochondrial matrix, is activated and transferred to the inner membrane of mitochondria, where it can bind F1F0-ATP synthase (mitochondrial complex enzyme V) [43–45] to promote opening of the mPTP. An abnormal increase in the expression or acetylation of CypD leads to excessive and irreversible opening of the mPTP [24], resulting in mitochondrial stress [25]; thus, CypD is considered the key regulator of mitochondrial stress [23]. Our previous study confirmed that CypD knockout alleviated high-fat diet-induced hepatic mitochondrial stress [26]. Our results showed that ADSCs reduced the increased expression of CypD induced by HG + AGEs in hepatocytes (Additional file 2: Fig. S2d). In summary, these in vitro results suggest that the ADSC supernatant suppressed hepatic mitochondrial stress induced by HG + AGEs.

Liver function is highly dependent on the production of ATP by mitochondria due to its role in biosynthesis and detoxifying properties. Due to the significant role of mitochondrial stress in liver injury and based on the above results, we speculated that ADSCs alleviate liver injury by ameliorating mitochondrial stress and dysfunction.

### Mechanistic exploration of ADSC-induced amelioration of liver injury

#### The suppressive effect of ADSCs on mitochondrial stress is related to apoptosis

**Fig. 5:**
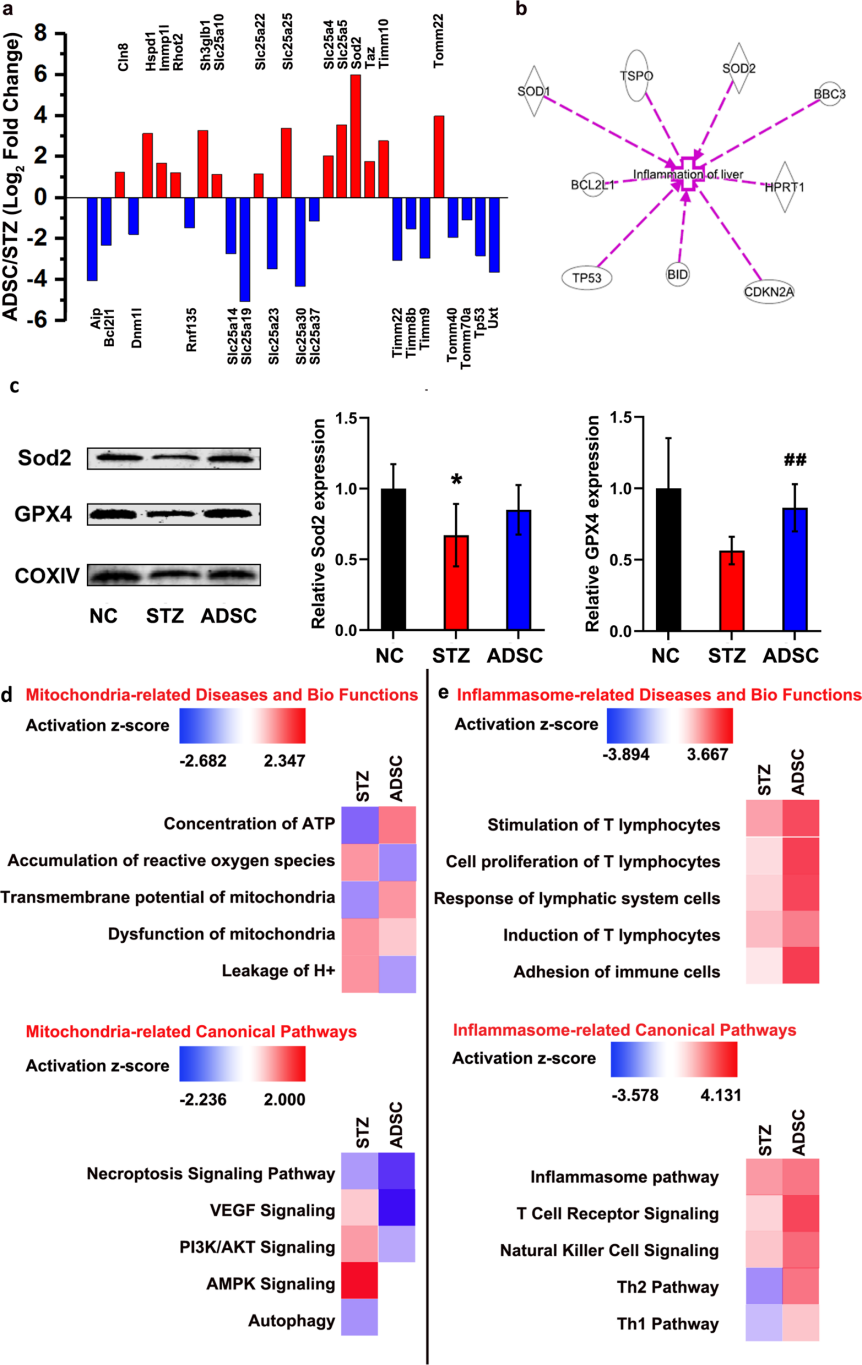
ADSCs attenuate liver injury by inhibiting apoptosis and then alleviating inflammation. **a** The DEGs in the ADSC group compared with the STZ group by mitochondria PCR array. **b**, **d**, **e** Ingenuity Pathways Analysis (IPA). **b** IPA network analysis indicated that mitochondria-related alterations can cause liver inflammation via Bcl2l1, Sod1, Sod2, etc. **c** Sod2 and GPX4 protein expression in the rat livers of three groups. *p < 0.05 versus the NC group, ##*p* < 0.01 versus the STZ group. **d** Disease, biofunctional and canonical pathway analyses of mitochondria. **e** Disease, biofunctional and canonical pathway analyses of the inflammasome. DEGs in these processes were found to be significantly changed in the ADSC group (**d**, **e**). Red represents upregulation, and blue represents downregulation

To explore the mechanism by which ADSCs inhibit mitochondrial stress and dysfunction, we used a PCR array to detect the expression of mitochondria-specific genes in the livers of diabetic rats. Genes were considered up- or downregulated if the average fold change in expression in the ADSC group compared to the STZ group was 2 or above (*p* < 0.05). Of the 84 genes examined, changes in mRNA levels were detected in 30 genes: 14 were upregulated and 16 were downregulated in the ADSC group compared to the STZ group. Compared with the STZ group, the ADSC group showed significant changes in the expression of genes involved in apoptosis, small molecule transport and mitochondrial membrane translocation, and among these genes, the expression of the representative genes Sod2, bcl2l1, Slc25a and Tomm40 was significantly changed. The DEGs are listed in Fig. 5a. To investigate possible biological interactions between the DEGs, datasets from genes with an altered expression profile were imported into the Ingenuity Pathway Analysis (IPA) tool. The necroptosis and apoptosis signaling pathways were the main canonical signaling pathways enriched in the ADSC group (Additional file 3: Fig. S3a), indicating that the suppressive effect of ADSCs against mitochondrial stress is related to the downregulation of necroptosis and apoptosis.

#### ADSCs attenuate liver injury by inhibiting apoptosis and then alleviating inflammation

Apoptosis and mitochondrial stress can cause tissue injury in T1D through a variety of pathways that may be related to inflammasomes. Activation of inflammasomes by islet amyloid polypeptide, hyperglycemia or autophagy insufficiency can lead to pancreatic β-cell dysfunction or apoptosis in T1D [46]. Inflammasomes can induce T1D by regulating endoplasmic reticulum stress-mediated inflammation and apoptosis in adipose tissue [47]. Mitochondrial stress can be caused by mitochondrial DNA lesions, which trigger the inflammatory response through Toll-like receptor 9 and inflammasome pathways to further exacerbate hepatocellular damage [48]. However, the specific roles of mitochondria and inflammasomes in T1D liver injury remain unclear. We next detected inflammasome-related gene expression in the livers of diabetic rats. We found that the expression of genes related to negative inflammasome regulation and signaling downstream of NOD-like receptors differed in the ADSC group compared with the STZ group, as shown by analysis with a rat inflammasome PCR array (Table 1), and IPA also found that some canonical pathways were enriched in DEGs in the ADSC group (Additional file 3: Fig. S3b). Genes associated with inflammation of the liver are shown in Additional file 4: Fig. S4, and half of these genes are involved in signaling downstream of inflammasomes or NOD-like receptors (shown in Table 1). These results suggest that ADSCs suppress liver inflammation through NOD-like receptor-related inflammasomes, which may be an important mechanism to attenuate liver injury.

**Table 1:** Representative up- and downregulated genes in ADSC versus STZ groups by an inflammasome PCR array

Catalog	Gene name	Change
Inflammasomes		
	Nlrp1a	↑↑↑
	Nlrp3	↑↑
	Pycard	↓
	Naip6	↓
Negative regulation of inflammasomes		
	Bcl2	↑↑
	Bcl2l1	↑↑
	Cd40lg	↑↑↑
	Ctsb	↑
	Hsp90aa1	↑↑
	Hsp90ab1	↑↑
	Mefv	↑
	Tnfsf11	↑↑
	Tnfsf4	↑↑↑
	Hsp90b1	↓↓↓
	Tnfsf14	↓
Signaling downstream of inflammasomes		
	Ifng	↑↑
	Il12a	↑↑↑
	Irak1	↑↑
	Mok	↑↑
	Panx1	↑↑
	Ptgs2	↑↑
	Ripk2	↑↑
	Txnip	↑↑↑
	Myd88	↓↓
	Il12b	↓↓
	Il33	↓
Signaling downstream of NOD-like receptors		
	Birc3	↑
	Ccl2	↑↑
	Chuk	↑↑
	Ikbkb	↑↑
	Irf2	↑
	Irf3	↑
	Irf4	↑↑
	Mapk11	↑↑
	Mapk14	↑↑
	Mapk3	↑↑
	Mapk8	↑
	Nfkb1	↑↑↑
	Nfkbia	↑
	Nfkbib	↑↑
	Rela	↑
	Ripk2	↑↑
	Tirap	↑↑↑
	Traf6	↑↑
	Xiap	↓
	Irf1	↓
	Irf5	↓↓
	Irf6	↓↓
	Map3k7	↓
	Mapk13	↓
	Tab1	↓
	Tab2	↓↓
	Ikbkg	↓↓
NOD-like receptors		
	Nlrp5	↑↑↑
	Nod2	↑
	Nlrp6	↓↓

Gene expression that was significantly increased or decreased was presented. The variations in gene expression between the ADSC and STZ groups are indicated by arrows, and fold-change values greater than 2 are shown

To identify the specific relationship between mitochondria and inflammasomes, IPA network analysis was conducted, and the results indicated that mitochondria-related alterations can cause liver inflammation via Bcl2l1, Sod1, Sod2, Tspo, Bbc3, Hprt1, Bid, Cdkn2a and Tp53 (Fig. 5b). Moreover, a prominent decrease of Sod2 and Glutathione Peroxidase 4 (GPX4), as the peroxidative repressors [49, 50], was observed in the liver of the STZ group, and ADSC treatment reduced this trend (Fig. 5c). It demonstrated that ADSC can partly improve the disruption of mitochondrial function in the liver. When we examined mitochondria-related diseases and biofunctions, DEGs in the process of ATP concentration, ROS accumulation and mitochondrial transmembrane potential were found to be significantly changed in the ADSC group (Fig. 5d). This result suggests improved mitochondrial function, which is consistent with our results in Fig. 4 and Additional file 2: Fig. S2. In addition, analysis of mitochondria-related canonical pathways showed that ADSCs affect VEGF, PI3K/AKT, AMPK signaling, autophagy and the necroptosis signaling pathway through changes in mitochondria (Fig. 5d). We further explored the mechanisms by which the inhibition of mitochondrial stress by ADSCs ameliorates liver injury. The results suggested that ADSCs attenuate liver injury and stimulate liver regeneration by altering the proliferation and activation of T lymphocytes, implying that this process is involved in the immune response (Fig. 5e). Representative processes and pathways are shown in Fig. 5d and e.

## Discussion

We found that ADSC injection improved liver function in rats, thereby alleviating oxidative stress and injury to the liver. Transplants of mesenchymal stromal cells (MSCs) are usually selected for clinical trials of T1D treatments [51, 52]. Although intrahepatic transplantation, which is commonly used, involves less immune rejection, it also causes some adverse reactions, such as MSC embolization [53]. Research has shown that MSC injection via the tail vein can mitigate pancreatic injury, hepatic lipid deposition, glomerular sclerosis and other lesions in rats [54], but ADSCs are widely applied because they are abundant, easily obtained and autologous in origin [55–57]. ADSC injection via the tail vein to rodents ① reduced serum ALT and AST levels and mortality [10] and ② increased hepatic Akt and ERK1/2 phosphorylation and serum albumin levels [11], suggesting that ADSCs have a therapeutic effect on acute liver failure and can promote liver regeneration. Our results confirmed that ADSC injection alleviated liver injury in patients with T1D, and these results are of great significance for early intervention and the delay of liver injury in T1D. However, further studies will be conducted to observe whether ADSCs in the liver can effectively repair and regenerate liver parenchyma in the long term for the treatment of chronic liver disease. Solving this problem will improve the application prospects of stem cell injection, and stem cell transplantation will no longer be an indispensable option for patients.

A study showed that only a limited number of umbilical cord-derived mesenchymal stem cells (UC-MSCs) were found in the liver, kidney and pancreas after stem cells were injected via the tail vein [54]. The therapeutic function of stem cells is dependent on not only local effects but also the indirect effects of immune regulatory factors or cytokine secretion [58]. When differential hepatic gene expression in the ADSC group compared to the STZ group was analyzed by RNA-seq, we found significant differences in the expression of cytokines and genes related to energy reserve, metabolic processes and the regulation of DNA-templated transcription in response to stress. The increased expression of VEGFα suggested that ADSC treatment promoted hepatic cell proliferation and angiogenesis, which together help to alleviate the local hypoxia that develops following injury [59].

The differential expression of FGF21 and TGF-β also suggested that ADSCs regulate liver injury and fibrosis. FGF21 was found to not only improve liver insulin sensitivity [60] and suppress fatty liver [61] but also reduce liver damage caused by long-term ethanol intake [62]. Furthermore, FGF21 secreted by stem cells enhanced the recovery of liver structures in a T2D mouse model by controlling the expression of insulin and SREBP [63]. We found that the expression level of FGF21 in the livers of rats in the STZ group was decreased compared with that in the NC group, implying that liver injury had occurred in the STZ group. Disordered intracellular energy metabolism is closely related to mitochondrial stress and mitochondrial dysfunction, and mitochondria are the main sites of ROS production. ROS induce hepatic stellate cells (HSCs) to secrete TGF-β by upregulating the nuclear transcription factor KLF6 [64] and activate the transformation of HSCs into myofibroblasts [65]. Furthermore, TGF-β activation is related to mitogen-activated protein kinase (MAPK) and Akt signaling [34]. The results of our study suggest that ADSCs improve mitochondrial function by regulating mPTP opening, which may be the underlying mechanism by which ADSCs ameliorate liver injury.

Diabetes is associated with mitochondrial dysfunction and chronic inflammation characterized by inflammasome activation and the release of proinflammatory mediators. Inflammasomes also play an important role in liver lesions; for example, nod-like receptor family pyrin domain containing 3 (NLRP3), the best known inflammasome, and sirtuins affect liver function in diabetes [66], but a mechanistic assessment is still needed. Our results also confirmed that many types of inflammasomes are associated with inflammation of the liver.

We wondered whether the effect of ADSC treatment in ameliorating mitochondrial stress is related to inflammasomes. To clarify the inherent relationship between mitochondria and inflammasomes, IPA was conducted, and the results indicated that ADSC treatment may affect downstream pathways such as the AMPK signaling, autophagy and necroptosis signaling pathways through changes in mitochondria. For example, the factors Bcl2l1, Sod2 and Tp53 were found to cause inflammation in the liver, implying that ADSCs attenuate liver injury in T1D by inhibiting apoptosis and alleviating inflammation. Mitochondrial stress and lesions can promote cell death, liver fibrogenesis and inflammation in the development of liver diseases by NLRP3 inflammasome and IL-1β activation [67]. Additionally, mitochondria-targeted antioxidants were found to improve liver inflammation and fibrosis in cirrhotic rats by preventing apoptosis [68]. Mitochondrial dysfunction is the primary cause of oxidative stress and inflammation in NAFLD, which can lead to the peroxidation of phospholipids and fibrogenesis [69]. Moreover, our results provide additional evidence of the mechanism by which mitochondrial stress can induce liver inflammation and thereby consequent liver injury and clarify the role of ADSCs. Therefore, ADSC treatment may represent a promising strategy for the prevention and treatment of liver injury.

Acute liver injury is characterized by inflammatory infiltration, the amplification of which can be reduced by inhibition of neutrophil-derived matrix metalloproteinases, neutralization of chemokines and inactivation of macrophages, thereby preventing injury [70]. In addition, the liver is an immunological organ, and lymphocytes in the liver are important for liver injury prevention. The liver lymphocyte population is enriched in unconventional T cells named natural killer T (NKT) cells, which play critical roles in first-line immune defense against invading pathogens and modulation of liver injury. Another kind of hepatic lymphocyte, NK cells, can mediate liver injury by balancing the local production of proinflammatory (Th1) and anti-inflammatory (Th2) cytokines upon activation through their activating and inhibitory receptors [71]. Activation of T cell receptor signaling can induce T cell proliferation, which is consistent with our IPA results in the ADSC group. Moreover, T cell proliferation and differentiation to form effector T cells may exacerbate liver injury in Con A-induced hepatitis [72]. However, different T cell subsets play different roles in liver injury, and our results suggested that ADSCs may mitigate liver injury by enhancing anti-inflammatory Th2 cells. The recent article demonstrated that MSCs from fetal liver have the advantage over other sources for their immunosuppressive, immunomodulatory and Foxp3 + T reg induction capacity [73]. The influences of MSCs on T regs could represent an important element of the therapeutic effects of MSCs for the treatment of immune-related disorders and transplantations.

Progressive liver fibrosis is the ultimate process for many liver injuries. Liver fibrosis is a dynamic process, and the most effective antifibrosis strategy is to cure the early, underlying stage before it progresses to advanced fibrosis. New therapeutic targets have been discovered, and interferon-γ, peroxisome proliferator-activated receptor γ ligands, angiotensin II antagonists and others have been used for clinical treatment, but their effects are not satisfactory [6].

ADSCs can repair damaged tissues, promote cell regeneration, regulate immunity and suppress inflammation, effects that play an important role in the treatment of liver injury and fibrosis. When ADSCs were cultured in vitro, a variety of cytokines were found in the ADSC supernatant. In addition to VEGF, TGF and FGF, as mentioned above, hepatocyte growth factor (HGF), placental growth factor (PGF), insulin-like growth factor (IGF), angiopoietin (Ang) and other cytokines were found in the ADSC supernatant. We observed the protective effect of ADSC supernatant on hepatocytes and found that ADSCs could ameliorate apoptosis and mitochondrial stress induced by HG and AGEs. Researchers have suggested that ADSC supernatant helps somewhat protect tissue from reperfusion injury, but the use of ADSC supernatant was not as effective as the local transplantation of ADSCs to the tissue [74]. However, the key question is whether implanted ADSCs can survive in tissues over the long term and whether their therapeutic effect is attributable to differentiation or a paracrine mechanism [75]. Thus, infusion of ADSC supernatant could be a new idea to treat tissue injury.

## Conclusions

In summary, ADSCs could attenuate diabetic liver injury by inhibiting mitochondrial stress and alleviating inflammation. It is important for early intervention in liver injury and for delaying the development of liver lesions in patients with T1D.

## Supplementary Information

**Additional file 1: Fig. S1.** Volcano plot depicting RNA-seq data from three groups

**Additional file 2: Fig. S2.** Effects of ADSCs on cellular apoptosis and mitochondrial stress in vitro. L02 cells were cultured with HG (25 mM) and AGEs for the indicated time, and ADSC supernatant was added to check its protective effects. (a) FCM. Cells were treated with HG and 100 µg/ml or 200 µg/ml AGEs for 48 h. (b) JC-1 staining (20X). Carbonyl cyanide 3-chlorophenylhydrazone (CCCP) was used as a positive control. The cells were treated for 24 h to detect MitoSox Red staining (c, 40X) and CypD protein expression (d). a: Control group, b: HG+AGEs (100 µg/ml) group, c: HG+AGEs (100 µg/ml)+ADSC group, d: ADSC group. **p<0.01 versus the control group and ##p<0.01 versus the HG+AGE group

**Additional file 3: Fig. S3.** IPA summary of ADSCs effects. Representative pathway list of mitochondria (a) and inflammasome (b) PCR arrays. Each histogram is a particular canonical pathway. The size of the histogram is correlated with increasing overlap significance

**Additional file 4: Fig. S4.** Inflammasome-related genes most associated with inflammation of the liver in IPA

## Data Availability

The data that support the findings of this study can be obtained from the corresponding author upon reasonable request.

## Abbreviations

T1D: Type 1 diabetes mellitus
ADSC: Adipose-derived stem cell
STZ: Streptozotocin
RNA-seq: RNA sequencing
FGF21: Fibroblast growth factor 21
TGF-β: Transforming growth factor β
TNF-α: Tumor necrosis factor α
mPTP: Mitochondrial permeability transition pore
T2D: Type 2 diabetes mellitus
NC: Normal control
ALT: Alanine aminotransferase
AST: Aspartate aminotransferase
ALP: Alkaline phosphatase
TG: Triglyceride
GGT: γ-Glutamyl transpeptidase
TC: Total cholesterol
H&E: Hematoxylin–eosin
DEGs: Differentially expressed genes
GO: Gene ontology
KEGG: Kyoto Encyclopedia of Genes and Genomes
VEGF-α: Vascular endothelial growth factor
AGEs: Advanced glycation end products
TSH: Thyroid-stimulating hormone
TEM: Transmission electron microscopy
ROS: Reactive oxygen species
HG: High glucose
FCM: Flow cytometry
IPA: Ingenuity Pathway Analysis
GPX4: Glutathione Peroxidase 4
MSCs: Mesenchymal stromal cells
UC-MSCs: Umbilical cord-derived mesenchymal stem cells
HSCs: Hepatic stellate cells
MAPK: Mitogen-activated protein kinase
NLRP3: Nod-like receptor family pyrin domain containing 3
NKT: Natural killer T
HGF: Hepatocyte growth factor
PGF: Placental growth factor
IGF: Insulin-like growth factor
Ang: Angiopoietin
