# Supplementary figures and images for: Adipose-derived stem cells alleviate liver injury induced by type 1 diabetes mellitus by inhibiting mitochondrial stress and attenuating inflammation
Source: Stem Cell Res Ther. 2022 Apr 1;13:132. doi: 10.1186/s13287-022-02760-z (PMC8973806; doi:10.1186/s13287-022-02760-z)

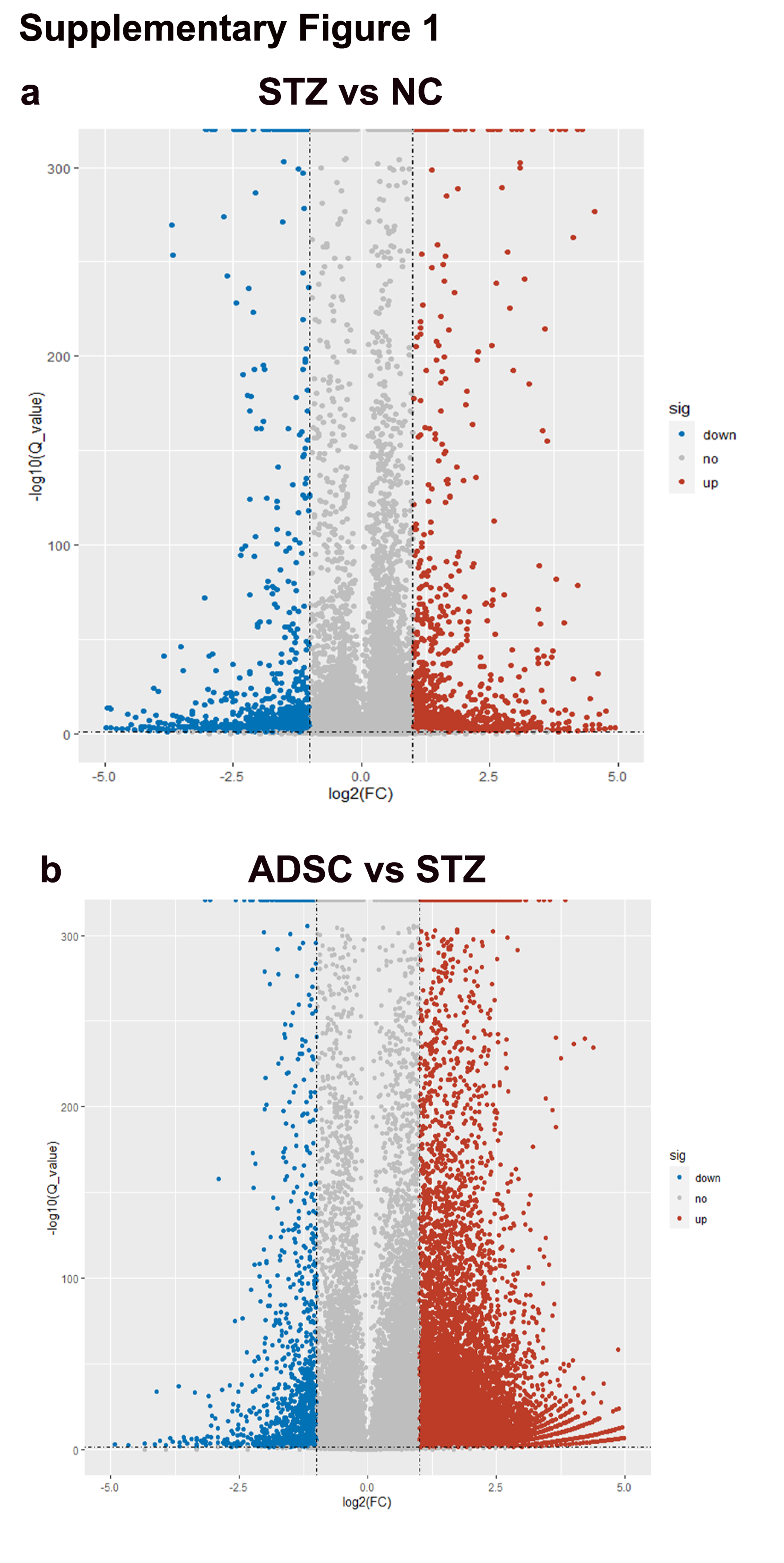

Supplement: Supplementary file 1 — Additional file 1: Fig. S1. Volcano plot depicting RNA-seq data from three groups [file 13287_2022_2760_MOESM1_ESM.tif]

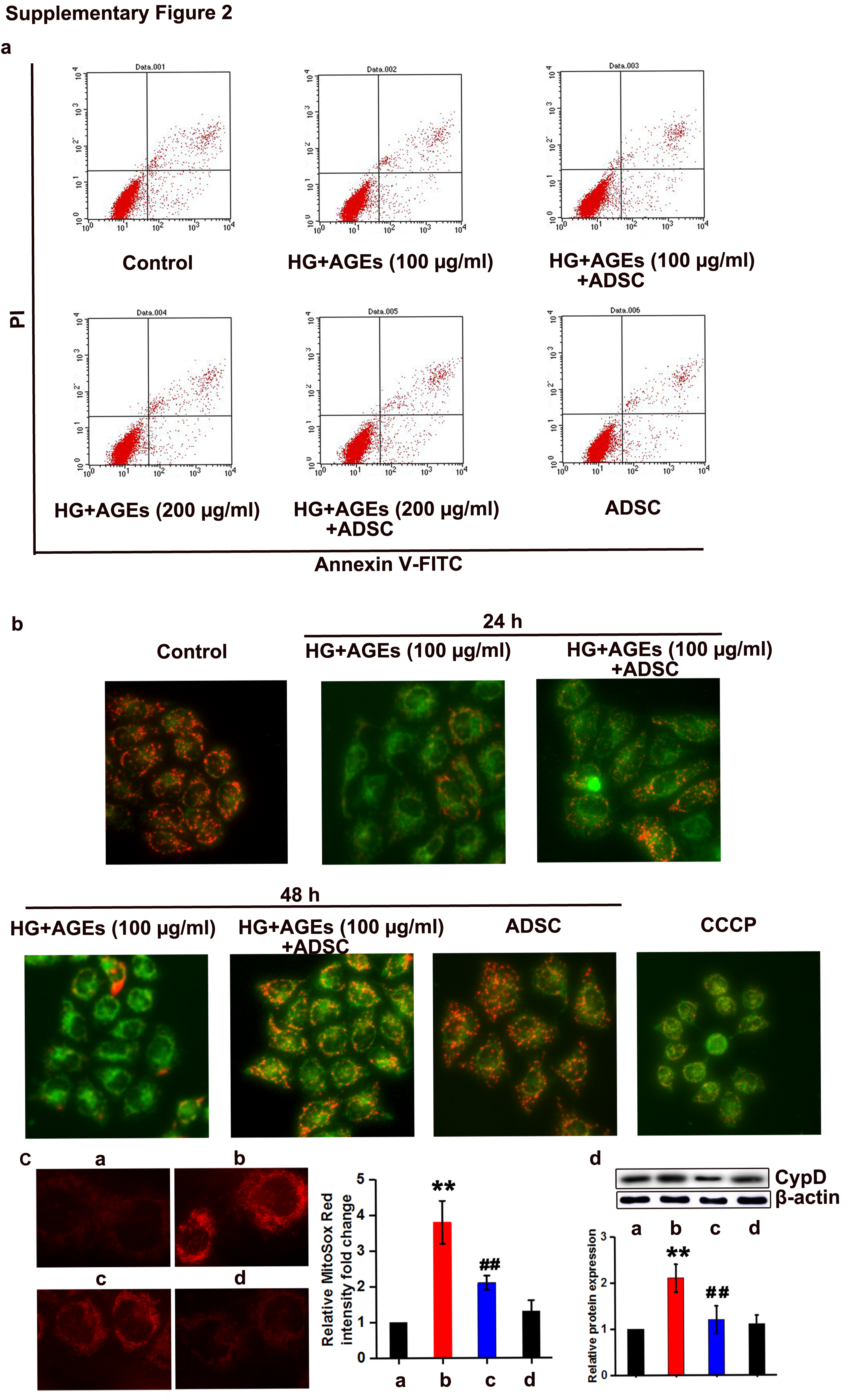

Supplement: Supplementary file 2 — Additional file 2: Fig. S2. Effects of ADSCs on cellular apoptosis and mitochondrial stress in vitro. L02 cells were cultured with HG (25 mM) and AGEs for the indicated time, and ADSC supernatant was added to check its protective effects. (a) FCM. Cells were treated with HG and 100 µg/ml or 200 µg/ml AGEs for 48 h. (b) JC-1 staining (20X). Carbonyl cyanide 3-chlorophenylhydrazone (CCCP) was used as a positive control. The cells were treated for 24 h to detect MitoSox Red staining (c, 40X) and CypD protein expression (d). a: Control group, b: HG+AGEs (100 µg/ml) group, c: HG+AGEs (100 µg/ml)+ADSC group, d: ADSC group. **p<0.01 versus the control group and ##p<0.01 versus the HG+AGE group [file 13287_2022_2760_MOESM2_ESM.tif]

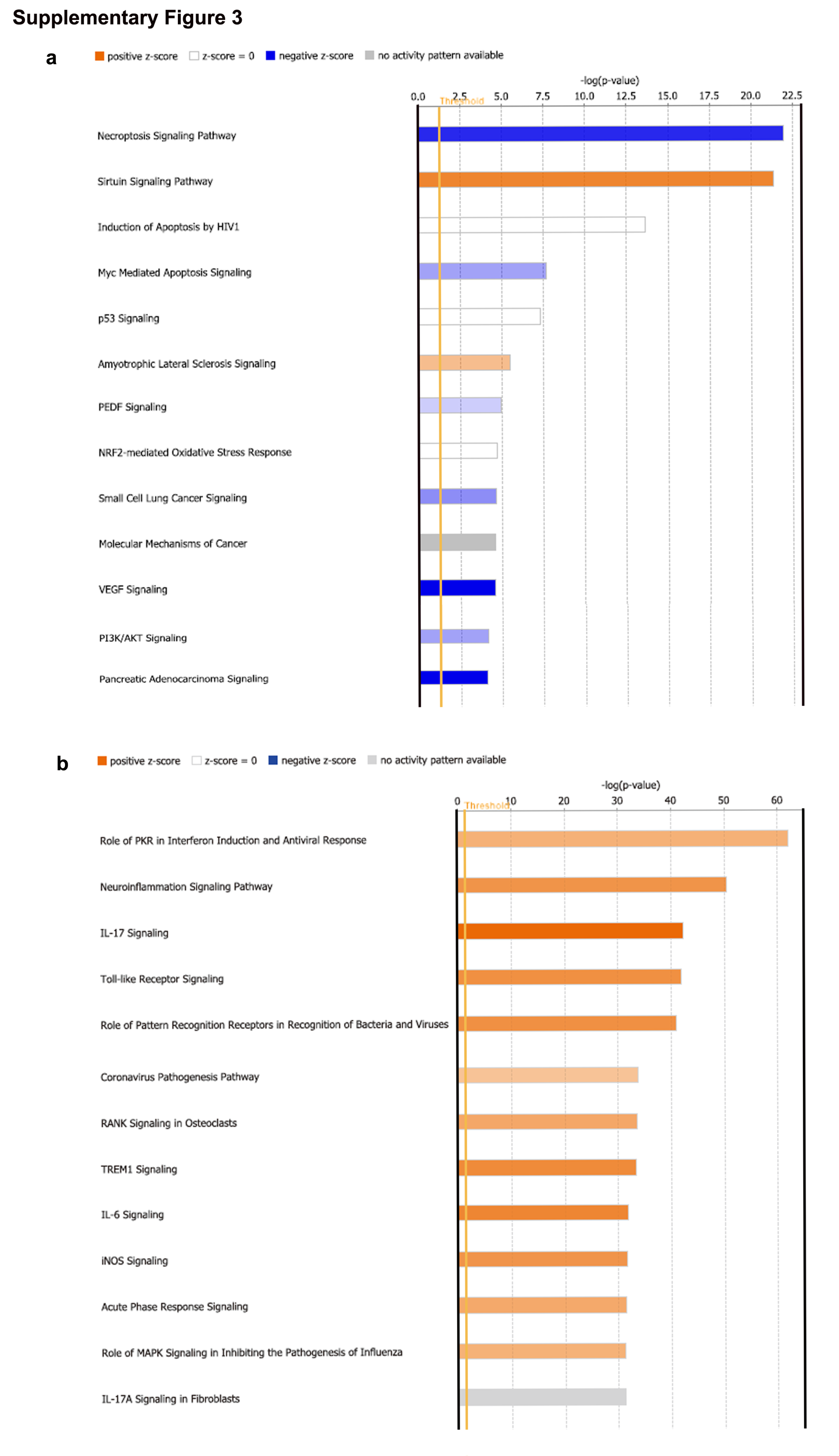

Supplement: Supplementary file 3 — Additional file 3: Fig. S3. IPA summary of ADSCs effects. Representative pathway list of mitochondria (a) and inflammasome (b) PCR arrays. Each histogram is a particular canonical pathway. The size of the histogram is correlated with increasing overlap significance [file 13287_2022_2760_MOESM3_ESM.tif]

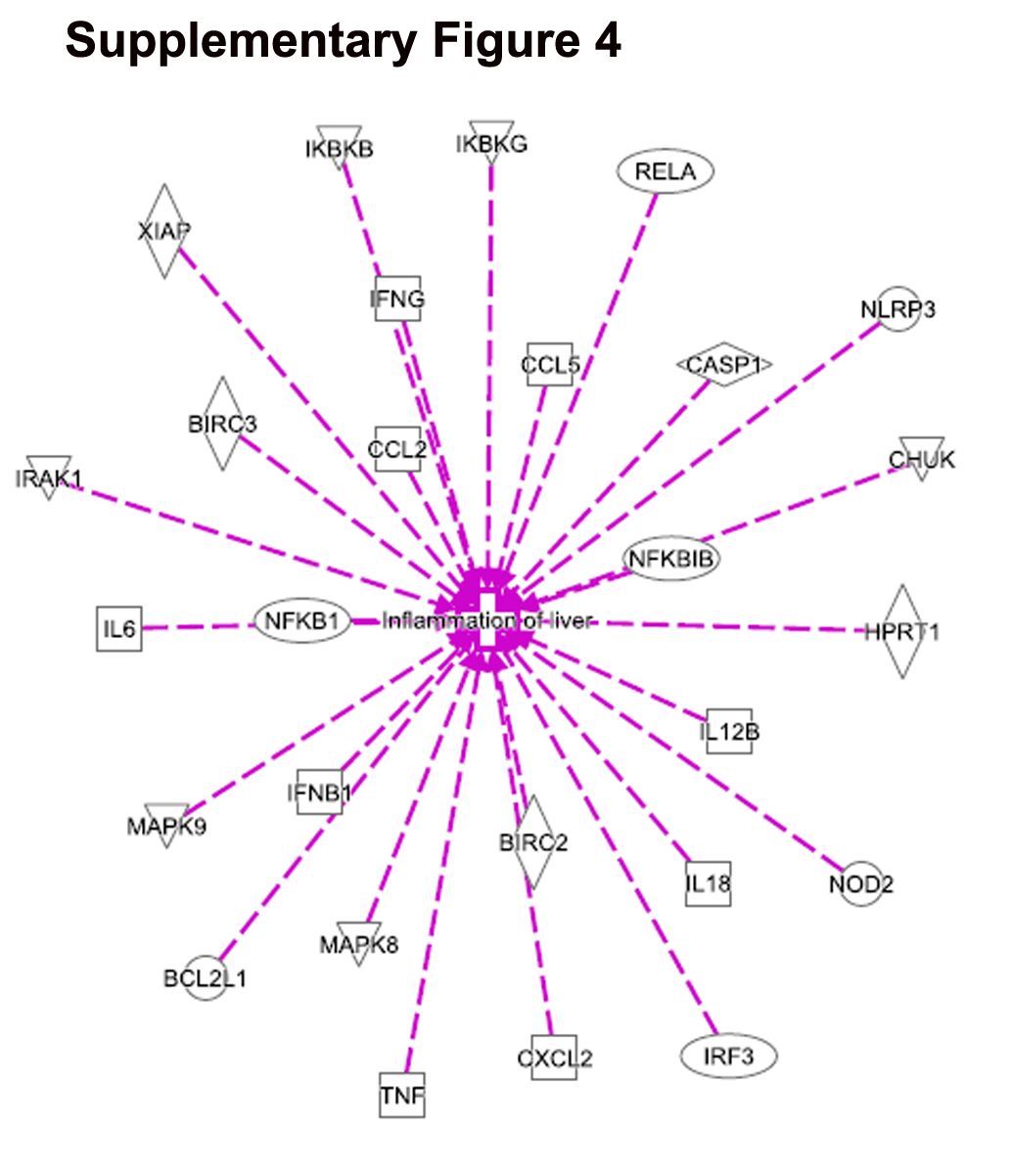

Supplement: Supplementary file 4 — Additional file 4: Fig. S4. Inflammasome-related genes most associated with inflammation of the liver in IPA [file 13287_2022_2760_MOESM4_ESM.tif]
